# Expression of cannabinoid 1 and, 2 receptors and the effects of cannabinoid 1 and, 2 receptor agonists on detrusor overactivity associated with bladder outlet obstruction in rats

**DOI:** 10.1186/s12894-017-0313-4

**Published:** 2017-12-29

**Authors:** Sung Dae Kim, Kang Jun Cho, Joon Chul Kim

**Affiliations:** 1 Department of Urology, Graduate School of Medicine, Jeju National University, Jeju, South Korea; 2 Department of Urology, Bucheon St. Mary’s hospital, College of Medicine, The Catholic University of Korea, Sosa-Ro 327, Wonmi-gu, Bucheon-si, Gyeonggi-do, Seoul, 14647 South Korea

**Keywords:** Bladder outlet obstruction, Cannabinoid, Overactive, Receptor, Urinary bladder

## Abstract

**Background:**

This study investigated changes in the expression of cannabinoid (CB) receptors and the effects of CB1 and CB2 agonists on detrusor overactivity (DO) associated with bladder outlet obstruction (BOO) in rats.

**Methods:**

Male Sprague Dawley rats were randomly assigned to four groups (*n* = 10) in each group. The control group comprised sham-operated rats. A animals in the BOO, CB1 agonist and CB2 agonist groups all underwent BOO surgery. Three weeks postoperatively, cystometrography (CMG) was performed on all rats. After confirming the presence of DO in the CB1 and CB2 agonist groups, a CB1 agonist (WIN 55,212–2) and a CB2 agonist (CB65) were instilled intravesically, and CMG was repeated. CMG parameters, including the contraction interval (CI) and contraction pressure (CP) were then analyzed. The bladders of rats in all four groups were excised following CMG. Immunofluorescence staining and Western blotting were performed to localize CB1 and CB2 and measure their expression levels in the urothelium and detrusor muscle.

**Results:**

The CI was significantly longer and the CP was significantly lower in the CB1 agonist group than in the BOO group. CI and CP in the CB2 agonist group showed the same results. CB1 receptor immunofluorescence staining signals and immunoreactive bands in Western blotting were increased in the BOO group compared with results in the control group. Similarly, results for the CB2 receptor were also increased in the BOO group, although this difference was not significant. The CMG parameters in the BOO group were significantly improved by the inhibitory effects of CB1 and CB2 agonists on BOO-associated DO. The expression of CB1 was significantly increased in the urothelium and detrusor muscle in BOO-associated DO, but no significant change in CB2 expression was observed.

**Conclusions:**

CB1 and CB2 receptors, especially CB1, play a role in the pathophysiology of BOO-associated DO, and could serve as therapeutic targets.

## Background

Detrusor overactivity (DO) seen in patients with overactive bladder (OAB) symptoms appears accompanied with bladder -outlet obstruction (BOO) in approximately 52% of cases [1]. The main causes of DO have been suggested to be associated with functional changes in the urothelium of the bladder. The importance of the urothelium as a physicochemical organoleptic cell layer has been shown to be significant because of the associations among urothelium, sensory neurons and smooth muscle cells, therefore, the development of a new drugs based on these interrelationships among in progress at an active area of research. Various receptors of the urothelium in the bladder, such as cannabinoid, acetylcholine, transient receptor potential vanilloid 4, and calcium-activated potassium channel receptors, mediate the pathophysiology of DO [2–6].

Cannabinoids, chemical components of marijuana, have been used to treat pain and vomiting for a long time. In recent years, cannabinoid receptors were found in the urothelium and detrusor muscle in the bladder, and it is believed that they are closely related to pain and sensory neurotransmission [7]. In addition, beneficial effects have been associated with the cannabinoid receptors cannabinoid-1 (CB1) and cannabinoid-2 (CB2) [8], which are known to be expressed in the urothelium and detrusor muscle in the bladder. Activation of these receptors could modulate bladder afferent activity and the micturition reflex [9]. It has been reported that many patients with multiple sclerosis and OAB showed improved after the administration of cannabinoid-based extracts [10]. Results from an animal model of spinal cord injury with the administration of a CB agonist and antagonist show that CB receptors play a key role in neurogenic bladder dysfunction [11]. Therefore, CB1 and CB2 receptors may function in the urinary bladder to help control bladder function. Recently, experimental studies have shown that CB receptors appear to be involved in afferent signaling pathways. Specially, Gratzke et al. noted that in rats with partial BOO treated with a CB agonist, the ability to empty the bladder was preserved, while non-voiding contraction frequency was decreased compared to that in controls [12]. In addition, Walczak et al. showed that the co-localization of CB1 receptors and P2X3 receptors in the bladders of mice declined in response to mechanically evoked bladder afferent activity in the pelvic nerve after the intravesical administration of a CB agonist [13]. Our study was designed based on the research findings discussed above.

We investigated changes in the expression of CB1 and CB2 receptors and the effects of CB1 and CB2 agonists on DO associated with BOO in male rats.

## Methods

### Experimental animals

This study was carried out using 16-week-old male Sprague Dawley rats weighing 250–300 g (Orient Bio Co., Seongnam, Korea). The rats were housed at room temperature (20–26 °C) under a 12-hlight/dark cycle with unlimited access to food. 40 rats were randomly assigned to four groups. The control group (*n* = 10) comprised sham-operated rats. The remaining three groups were the BOO group (*n* = 10), CB1 agonist group (*n* = 10) and CB2 agonist group (*n* = 10), all of which underwent BOO surgery. We used WIN55,212–2, and CB65 as representative CB1 and CB2 receptor agonists, respectively. For the CB1 and CB2 agonist groups, drugs were instilled into the bladder of each rat.

### BOO procedure

Experimental rats were anesthetized using an intramuscular injection of 5 mg/kg xylazine and 15 mg/kg ketamine. A suprapubic midline incision was made in the lower abdomen of rats. The bladder neck and periurethral areas were dissected out. A 25-G needle sheath was inserted into the urethra, and the bladder neck was ligated using 3–0 silk, after which the sheath was removed. In the sham-operated control animals, the bladder neck was very loosely ligated so as to not cause any obstruction.

### Cystometrography (CMG)

CMG was performed on all rats 3 weeks postoperatively, when we confirmed the presence of DO in the all three groups undergoing BOO surgery. A 25-G needle connected to polyethylene tubing was inserted into the bladder dome. The tubing was connected to a pressure transducer and an infusion pump using 3-way stopcock. The bladder was emptied and then continuously filled with warm saline using a Harvard syringe pump. CMG parameters including contraction interval (CI) and contraction pressure (CP) were recorded using a polygraph apparatus (Grass 7D, Grass Institute Co., Quincy, USA). In addition, the bladder was emptied in the CB1 and CB2 agonist groups, and a penile clamp was used to impede urination. Then 50 μM/kg of a CB1 receptor agonist (WIN55,212–2) and 10 μM/kg CB2 receptor agonist (CB65) in 0.2 mL of saline was injected through a needle into the bladder of each rat in these two groups. Then, we repeated CMG 1 h after the administration of each drug, with the bladder filled with saline.

### Bladder collection and immunofluorescence staining

After CMG was completed, the bladders of all rats were excised, cut vertically and dissected under a microscope into the urothelium and detrusor muscle. Bladder sections were frozen in liquid nitrogen for further analysis. Using optimal cutting temperature solution and, polyethylene glycol, the frozen tissues were cut into 3-μm sections. The tissue sections were then rinsed with phosphate-buffered saline (PBS). To inhibit nonspecific immunofluorescence staining, slides were exposed to a blocking solution (1.5% normal goat serum, 1.5% normal horse serum, 1% bovine serum albumin, 0.1% Triton X-100 in PBS) at room temperature for 1 h. Immunolabelling was performed by incubating samples with antibodies against CB1 and CB2 (1:100 dilution, Abcam Ltd., Cambridge, UK) in PBS for 2 h at room temperature. The slides were rinsed three times with PBS and then incubated with the secondary antibody, Alexa Fluor 488-labelled goat anti-rabbit IgG (1:300 dilution, Molecular Probes, Eugene, USA) at room temperature for 1 h. After washing in PBS, the slides were stained with 4′,6-diamidino-2-phenylindole (Vector Laboratories, Burlingame, USA), and mounted for examination using light microscopy (BX50, Olympus, Tokyo, Japan). Staining was visualized using fluorescence microscopy, and digital images were captured. Because CB1 and CB2 receptor-expressing cells were stained green, their immunoreactivity was evaluated as the percentage of green-stained area in three random fields per image using an imaging analysis program (IMTi-Solution ver.10.1, IMTi-Solution Inc., Montreal, Canada).

### Western blotting

Western blotting was performed to compare the expression levels of CB and CB2 receptors in the urothelium and detrusor muscle layer in rats from each group. Frozen tissue samples were pulverized and then homogenized at 4°C using a Qproteome Mammalian Protein preparation kit (Qiagen, Hilden, Germany). The homogenates were centrifuged at 900 *g* for 20 min at 4°C. The pellets were discarded, and the supernatant was either used immediately or stored at −70°C. Total protein was measured using a Bradford dye-binding protein assay kit (Bio-Rad Laboratories, Hercules, USA) according to the manufacturer’s instructions, and the equivalent of 50 mg of total protein was loaded onto polyacrylamide gels with primary antibodies against CB1 and CB2 receptors. The samples were electrophoresed using a Mini-Protean 3 Cell system (Bio-Rad Laboratories) under constant voltage. After electrophoresis, the proteins were transferred to polyvinylidene fluoride membranes for 2 h at 4°C using a transblot semidry transfer cell (Mini-Protean 3Cell, Bio-Rad). Nonspecific binding sites were blocked by incubating the membranes with Tris-Tween-buffered saline (TBS-T) containing 10% skim milk and 0.1% Tween 20 for 1 h at 4°C. The membranes were then incubated overnight at 4°C with a biotinylated antibody against either the CB1 or CB2 receptor (1:5000 dilution in TBS-T, Abcam PLC). After washing with TBS-T, the membranes were incubated for 30 min at room temperature with goat anti-rabbit IgG horseradish peroxidase (1:5000 dilution) and then washed. Immunoreactive proteins were detected and visualized using a chemiluminescence reagent (ECL, Amersham International, Bucks, UK).

### Statistical analysis

All data were analyzed using Sigma Stat for windows (Version 3.0, SPSS Inc., Chicago, IL, USA). The data are expressed as the mean ± SEM. For comparisons among the groups, one-way analysis of variance (ANOVA) and Newman-Keuls multiple comparison tests were performed with *P* < 0.05 as an indication of statistical significance.

## Results

**Fig. 1:**
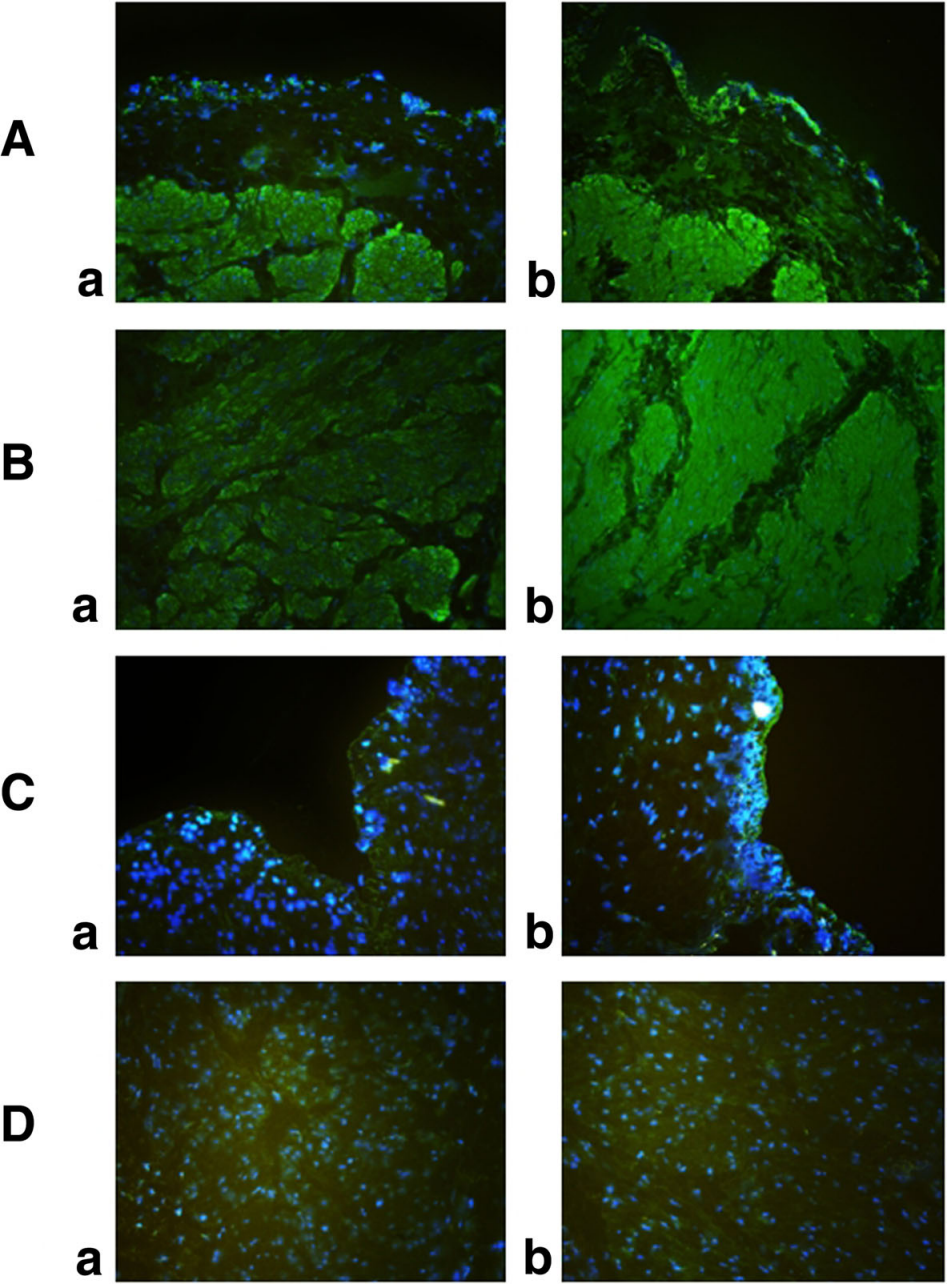
Immunofluorescence staining of CB1 and CB2 receptors. **a** and **b** show that there were significant increases in CB1 receptor immunoreactivity in the urothelium (*a*) and detrusor muscle (*b*) of rats in the BOO group. **c** and **d** show that there were no differences in CB2 receptor immunoreactivity in the urothelium (*a*) and detrusor muscle (*b*) between the control group and the BOO group. Green shows CB1 or CB2 receptor expressing cells and blue dots show DAPI staining of nuclei. BOO:bladder outlet obstruction. CB:cannabinoid

CMG showed that BOO group had a significantly higher CP (28.03 ± 3.94 cmH_2_O vs. 23.03 ± 3.07 cmH_2_O, *p* < 0.05) and significantly shorter CI (4.42 ± 0.76 min, vs. 10.52 ± 1.07 min, *p* < 0.05) than the control group, as shown in Table 1. Three weeks postoperatively, the CB1 agonist group had a significantly longer CI (8.61 ± 0.61 min vs. 4.42 ± 0.76 min, *p* < 0.05) and lower CP (19.88 ± 3.14 cmH_2_O vs. 28.03 ± 3.94 cmH_2_O, *p* < 0.05) than the BOO group, as did CB2 agonist group (CI 9.51 ± 0.55 min vs. 4.42 ± 0.76 min, *p* < 0.05; CP 23.76 ± 4.78 cmH_2_O vs. 28.03 ± 3.94 cmH_2_O, *p* < 0.05). Moreover, the immunofluorescence signal for CB1 receptors was significantly increased in urothelium and detrusor muscles of the BOO group compared with that of control group. The immunofluorescence signal for CB2 receptors was also increased in the BOO group, but this increase was not statistically significant (Fig. 1).

**Table 1:** Cystometrography results for the experimental groups

	Control group (*n* = 10)	BOO group (*n* = 10)	CB1 agonist group (*n* = 10)	CB2 agonist group (*n* = 10)	P value^a^		
					control vs BOO	BOO vs CB1 agonist	BOO vs CB2 agonist
Contraction Pressure Mean(SD)cmH_2_O	23.03 (3.07)	28.03 (3.94)	19.88 (3.14)	23.76 (4.78)	< 0.05	< 0.05	< 0.05
Contraction Interval Mean(SD) min	10.52 (1.07)	4.42 (0.76)	8.61 (0.61)	9.51 (0.55)	< 0.05	< 0.05	< 0.05

*BOO* bladder outlet obstruction, *CB* cannabinoid

^a^analysis of variance test showed significant difference in all 3 parameters among all 3 groups

**Fig. 2:**
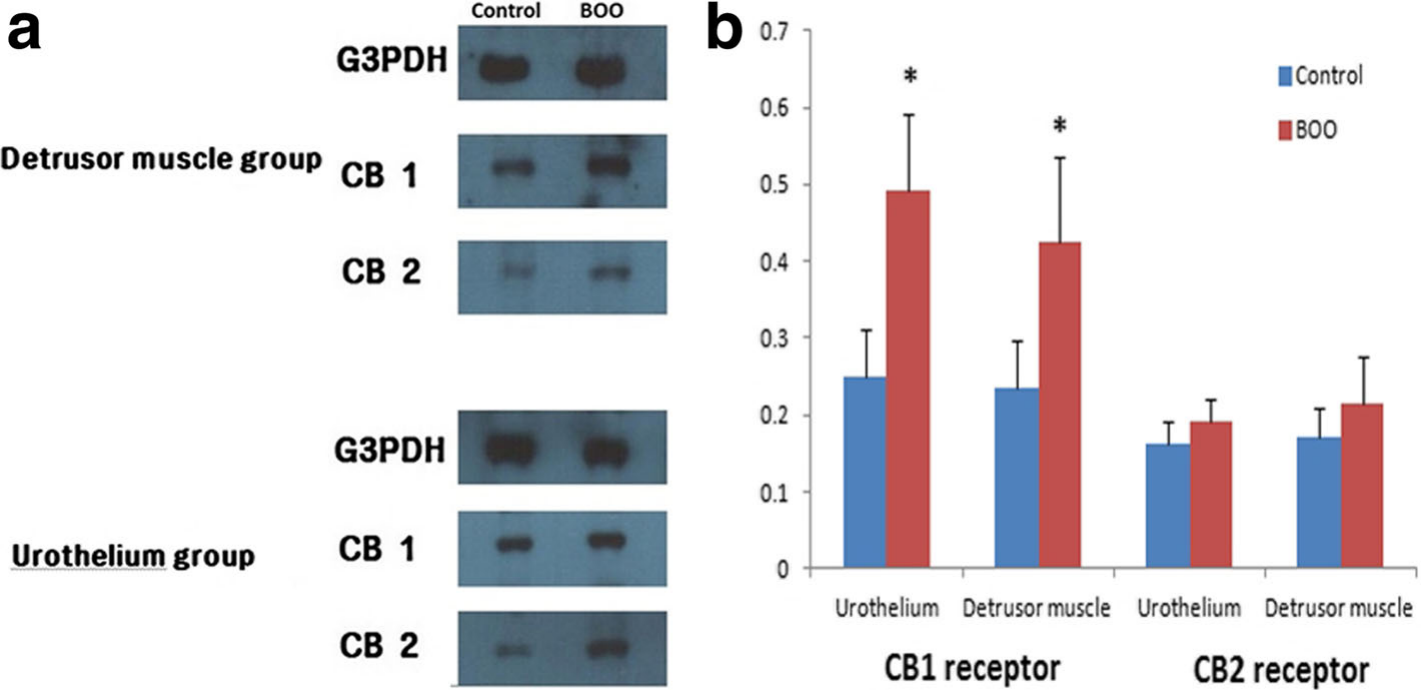
Comparison of CB1 and CB2 receptors expression levels from Western blot analysis between the control group and the BOO group. CB1 receptor expression was significantly increased in the BOO group compared to the control group (*p* < 0.05). Immunoreactive bands for the CB2 receptor were also increased in the BOO group, but the difference was not significant (*p* > 0.05) **a**: Western blotting **b**: densitometry results relative to G3PDH. Data are expressed as the mean ± SD. * < 0.05 compared with the BOO group. BOO:bladder outlet obstruction, G3PDH:glyceraldehyde-3-phosphate dehydrogenase, CB:cannabinoid

Immunoreactive bands from Western blotting indicated that the expression of both CB1 and CB2 receptors were present in the urothelium and detrusor muscle of rats in all groups. CB1 receptor expression was significantly increased in the BOO group compared with expression in the control group (*p* < 0.05). However, although the immunoreactive bands for the CB2 receptor were also increased in the BOO group, this increase was not statistically significant (*p* > 0.05) (Fig. 2).

## Discussion

The animal model used in this study, does not completely represent patients with OAB, but it has been demonstrated in several studies as the best animal model of OAB [14, 15]. The methods used here are based on previous finding, as discussed below. Hedlund et al. reported on CB1 and CB2 receptors, and showed that CB1 and CB2 agonists inhibit nerve-mediated contractions of the mouse bladder [16]. Representative CB1 agonists include, CP55244, WIN55,212–2, delta-9-tetrahydrocannabinol (THC), arachidonyl-2′-chloroethylamide (ACEA), and nabilone. CB2 agonists, CP55940, CB65. In this study, we used WIN55,212–2 and, CB65 as CB1 and CB2 agonists, respectively. Systemically administered cannabinoids can act at multiple sites, including the bladder and many sites in the central nerve system. Intravesical activation of CB1 does block Nerve Growth Factor-induced increased bladder activity [17]. In addition, according to Tyagi et al., CB1 and CB2 receptors have higher expression rates in the bladder urothelium, and bladder strips incubated for 15 min CB agonists show a decreased detrusor muscle contraction amplitude [8]. Therefore, we thought that intravesically instilling these drugs into the urothelium of bladder could affect detrusor contraction.

We interpret the experimental results of this study as follows. First, the CMG findings of a higher CP and shorter CI in the BOO group than in the control group provided clear evidence that DO was present in the BOO group. The results also showed that DO was significantly improved by the inhibitory effects of CB1 and CB2 receptor agonists. Second, the expression of the CB1 receptor was significantly increased in the urothelium and detrusor muscle in rats with BOO-associated DO, but no significant change in the expression of the CB2 receptor was observed. These findings suggest a key role for CB1 and CB2 receptors, particularly the CB1 receptor, in the regulation of bladder function in rats with BOO-associated DO. Many previous studies have shown that cannabinoids affect bladder DO in patients with OAB symptoms and CMG parameters reflecting sensory function, and they reduce the sensory activity of isolated tissues, and induce antihyperalgesia in animal studies of bladder inflammation [16]. Endocannabinoids relax the detrusor muscle during filling of the bladder by binding to CB1 and CB2 receptors, possibly modulating sensory afferent signals [18]. However, there are also contradictory results from previous studies of the expression and function of CB receptors, thus, the mechanism of this effect remains unclear.

In our study, both Western blotting and immunofluorescence showed that in BOO-associated DO, CB1 receptor expression in the urothelium and detrusor muscle was significantly increased, while CB2 receptor expression was not significantly changed. These results are consistent with those of Tyagi et al. [8], who suggested that both CB1 and CB2 receptors are expressed in the urothelium and detrusor muscle and that CB1 receptor expression was significantly higher than that of CB2 receptor. They also suggested that CB2 receptor expression did not increase at the onset of DO or an inflammatory response because CB2 receptors are mainly expressed in peripheral immune cells; our results support their hypothesis. In a study using CB1 receptor-knockout mice, Füllhase et al. reported that the pathophysiologic role of CB1 receptors, but not that of CB2 receptors, is related to central and peripheral nervous control of micturition [19, 20].

In contrast to these studies, there are reports that it is not CB1 receptors, but rather CB2 receptors that play an important role. For example, Gratzke et al. [21] reported that increased CB2 receptor expression, but not increased CB1 receptor expression, was present in the urothelium and sensory nerves, and that a CB2 agonist improved the micturition interval and threshold, suggesting that the CB2 receptor mediate afferent signals in the bladder. Another study reported that the CB2 receptor was upregulated in a rat model of bladder inflammation [22]. Lastly, it was recently reported by Bakali et al. [23, 24] that in both rat and human bladders, CB1 receptors were localized in the urothelium, detrusor muscle, and nerve fiber structures of the subendothelium, and have both pre- and post-synaptic inhibitory effects, whereas CB2 receptors were localized in the same sites in the human bladder, but only in the detrusor muscle in rat bladders, suggesting that CB2 receptors only have postsynaptic inhibitory effects.

The differences between our study and previous studies could be due to the different methodologies. In our study, we used intravenous ketamine and xylazine to anesthetize rats, whereas Gratzke et al. [21] used intraperitoneal administration of isoflurane and urethane and performed CMG on conscious rats. It has been reported that ketamine inhibits the micturition reflex [25].

This study had several limitations. First, we used one dose and type of agonist for CB1 and CB2 receptors, however, these doses and types may not produce the maximal effect on the bladder of rats. Definitive characterization of CB receptor expression in tissues depends on the availability of selective agonists and antagonists. Further studies are warranted to examine these receptors in the urothelium and detrusor muscle and to develop drugs targeting these receptors. Such studies would be helpful for regulating DO caused by BOO and to control OAB symptoms. Second, we did not measure residual urine volume or functional bladder capacity in the rat model used here. CB1 and CB2 agonists may reduce the resistance provided by external sphincter, thereby indirectly reducing bladder CP. Therefore, we can not exclude the possibility that reduction in CP was because of a decrease in urethral resistance after the administration of CB1 and CB2 agonists. Third, although we attempted to measure non-voiding contractions, we were unable to do so; in this study, CP and CI were measured at voiding. It is also possible that our conclusions are not valid for technical reasons. We considered that the changes in CP and CI suggested the presence of DO, and may therefore provide information regarding changes in bladder status related to DO. Fourthly, both immunofluorescence staining and Western blotting showed that CB2 receptor density was not significantly higher in the BOO group than in the control group. The cause to explain this discrepancy is not precisely known, but it is presumed that CB2 receptors are involved in the mechanisms discussed in this study but to a lesser degree than CB1 receptors. The factors underlying these differences were not revealed by the methods used here. However, there was a statistically significant difference in the control, CB1 agonist, and CB2 agonist group for CMG parameters relative to the BOO group (*p* < 0.05).

In addition, although treatment with CB1 and CB2 agonists can have beneficial effects on DO, they can also cause complications or side effects during long-term administration. However, our study did not reveal any serious complications. Complications may have been avoided because agonists were administered intravesically, which is a local injection rather than a systemic injection. A potential problem is that it may be difficult to determine the most appropriate method of administration and dosage of CB agonist in future clinical studies of human patients.

Ultimately, CB1 receptor expression was upregulated in the bladder in BOO-associated DO. A possible explanation for this may be feedback activation via decreased levels of endocannabinoid ligands. The concentration of endocannabinoids in the bladder, which have inhibitory effects, may decrease during BOO-associated DO.

## Conclusions

CMG parameters in the BOO group were significantly improved by the inhibitory effect of CB1 and CB2 receptor agonists on DO associated with BOO. The expression of CB1 was significantly increased in the urothelium and detrusor muscle in DO associated with BOO, but no significant change in CB2 expression was observed. The results of this study suggest that CB1 and CB2 receptors in the bladder, particularly CB1 receptors, play a significant role in the pathophysiology of BOO-associated DO, and could serve as diagnostic biomarker and therapeutic targets in this disorder.

## Abbreviations

BOO: Bladder outlet obstruction
CB: Cannabinoid
CI: Contraction interval
CMG: Cystometrography
CP: Contraction pressure
DO: Detrusor overactivity
G3PDH: Glyceraldehyde-3-phosphate dehydrogenase
PBS: Phosphate-buffered saline
